# Mr. Dunning, on Strangulated Hernia

**Published:** 1800-04

**Authors:** Richard Dunning

**Affiliations:** Plymouth Dock


### *To the Editors of the Medical and Phyſical Journal.*

GENTLEMEN,

THE incloſed having been well thought of by ſeveral of my medical acquaintance, to whoſe opinions I have always paid great deference, and drawn from Dr. Remmett ſome collateral remarks, which I conceive to be very valuable, and of which, at my particular requeſt, the Doctor has permitted me to make what uſe I judge proper; I am encouraged to offer it for inſertion in your much approved Journal; and at the ſame time, to deſire you will ſubjoin to it, Dr. Remmett's letter, as I know not how to diſpoſe of it better, than by giving it a place in a work which ſo generally obtains, and connecting it with the circumſtance that gave riſe to it. I am,

GENTLEMEN,

Your moſt obedient humble ſervant,

RICHARD DUNNING.

*Plymouth Dock,*

*February* 22, 1800.

---

DEAR SIR,

HAVING lately had a caſe of ſtrangulated Hernia under my management, the removal of which I attribute, in a great meaſure, if not wholly, to the exhibition of the Fox-glove, I wiſh to ſtate to you a few minutes of it, and to requeſt (if you judge it worth while) that you'll forward them to Dr. Ferriar, to whom I am of opinion, that every fact or obſervation ariſing from the uſe of this excellent remedy ſhould be addreſſed, as a well-earned acknowledgement of the juſt diſcrimination and great abilities, evinced by him in his late very valuable and diſpaſſionate communication to the medical world on that ſubject. And I am the more readily induced to aſk this of you, becauſe I know with how much earneſtneſs you are, at all times, and on all occaſions, embarked in promoting the beſt intereſts of the profeſſion; and becauſe alſo, I know that your attention is at preſent very particularly engaged in the inveſtigation and appreciation of this novel, or rather revived medicine. It may not, perhaps, be irrevelant to remark, that judging from what we know of the general œconomy of Providence, it is not very probable that this beautiful plant, which is almoſt every where, for ſeveral months in the year, challenging and even commanding our obſervation, by the luxuriance of an elegant and ſtately flower, ſhould have been given us merely as an ornament to our our fields and hedges — I believe it about to aſſume an additional and more important character.

A. B. between ſixty and ſeventy years old, of a ſtrong, hale conſtitution, had many years been ſubject to a large ſcrotal hernia, and had many times ſuffered ſymptoms of ſtrangulation for ten or twelve hours; but had hitherto, after a little reſt, without any medical aſſiſtance whatever, been himſelf able to replace it. When I was lately called to him, it had been down nearly thirty hours, though he had, as uſual, reſted himſelf, and had made more violent attempts than formerly, to reduce it. The tumour was large, hard, very tenſe, and extremely painful to the touch. He complained of exceſſive thirſt; and his pulſe was very full and ſtrong. I immediately bled him largely; and after having made long, though gentle, ineffectual efforts to reduce the hernia, ordered a clyſter to be injected as ſoon as poſſible, the parts to be conſtantly covered with ſeveral folds of linen cloth, wetted in a cold ſolution of ſal ammoniac and nitre in vinegar and water, and forbade every thing which could have the ſmalleſt tendency to keep up inflammation. Calling ſeveral hours afterwards, I was concerned to find that no advantages had been obtained from what had yet been done; I thought, indeed, every ſymptom aggravated: the tumour was not leſs tenſe, certainly more painful; the pulſe harder, the thirſt unabated, the heat and dryneſs of the ſkin extreme; and there was about him that reſtleſſneſs and anxious diſtreſs which we frequently obſerve in caſes of this nature.

As the ſecond attempt was as unſucceſsful as the firſt, I began to think very badly of his ſituation, and that nothing but an early operation would give my poor patient a chance of eſcape; when, *perhaps fortunately for him*, the Digitalis occurred to me: and I was inſtantly moſt forcibly ſtruck with the idea, that this was one of thoſe caſes, of all others, the moſt likely to be benefited by the uſe of it. I accordingly gave him, Pulv. digital. (the powder was moſt carefully prepared) gr. i. opii colat. gr. i. calomel gr. iv. together with a draught, in which were xx drops of the tincture of Fox-glove, made from the green leaves; and directed theſe remedies to be repeated every four or ſix hours. Soon after the firſt doſe, however, he became evidently eaſier; and very ſhortly after the ſecond, he fell into the moſt complete ſtate of relaxation ſhort of faintneſs, and into the moſt perfect calm, I ever remember to have ſeen. The tenſion of the general ſyſtem, and, along with it, that of the tumour, having ſo completely given way, I was enabled to reduce the contents of the latter by the moſt inconſiderable preſſure; and I had the great ſatisfaction to ſee my patient recover, in the courſe of a few days, without interruption.

That theſe moſt deſirable effects might have been entirely the conſequences of the opium and calomel, without an auxiliary, I cannot venture to deny. Be this, however, as it may, I muſt be allowed to ſay, that I have never hitherto obſerved any thing ſo deciſive from their uſe in ſimilar caſes; that therefore, I cannot help feeling myſelf under ſome obligations to the Fox-glove in this inſtance; and conſider it a duty not to ſuffer this caſe, though a ſolitary one, to paſs by unnoticed.

I am, Dear Sir,

At all times, with great reſpect,

Your moſt obedient humble ſervant,

RICHARD DUNNING.

*Plymouth Dock,*

*Feb.* 20, 1800.

To Dr. Remmett.

---

### *To Mr. DUNNING.*

My dear Sir,

YOU have very highly gratified me, by making me the inſtrument of conveying to Dr. Ferriar your Caſe of ſtrangulated Hernia, accompanied by ſentiments of reſpect for him, in which I heartily join. And I beg leave to offer you my unfeigned thanks for the very liberal teſtimony you have given to my poor endeavours to ſupport the credit of a profeſſion, whoſe true intereſts have indeed been long among the firſt wiſhes of my heart.

Your Caſe contains a moſt important ſuggeſtion, the reſult of very ſound judgment, both with reſpect to the diſorder and the remedy. Any more than yourſelf, I will not attempt to determine the exact degree of benefit which was produced by the Digitalis; but I have no doubt, that in the future treatment of colic, as well as in caſes of ſtrangulated hernia, whether attended with ſymptoms of conſiderable inflammation or not, as alſo in other inſtances of fixed ſpaſm, ſuch as locked jaw and the like, the Fox-glove will be found a moſt important aid. The atony, induced by it, is more complete than can be effected by any other means with which I am acquainted. I am,

My dear Sir,

With great regard,

Moſt truly, your's,

B. B. REMMETT.

*Plymouth,*

*Feb.* 21, 1800.

